# Ocular Hypotonia and Transient Decrease of Vision as a Consequence of Exposure to a Common Toad Poison

**DOI:** 10.1155/2020/2983947

**Published:** 2020-01-16

**Authors:** Renato Pejic, Marija Simic Prskalo, Josip Simic

**Affiliations:** ^1^University Clinical Hospital Mostar, Department of Ophthalmology, Bosnia and Herzegovina; ^2^Faculty of Health Studies, University in Mostar, Bosnia and Herzegovina

## Abstract

The common toad produces venom (bufotoxin) that is produced in the parotid gland of the toad as well as in the skin. This toxic compound is a potent inhibitor of Na^+^/K^+^-ATPase activity. Physiological effects of bufotoxin are similar to those of digitalis and cause increased heart rate and muscle contractions. Ocular toxicity was described. A 67-year-old female patient was admitted to the emergency service because of sudden vision loss and a burning sensation in both eyes after she had been exposed to the poison of a toad. Slit lamp examination showed conjunctival hyperaemia and signs of ocular hypotonia. Topical antibiotic treatment was administered, and after 24 hours, corneal oedema and ocular hypotonia were in remission. Inhibition of Na^+^/K^+^-ATPase is a well-known effect of the toad venom. Na^+^/K^+^-ATPase is a part of corneal endothelial cells, ciliary body, and iris, and its inhibition caused by exposure to bufadienolides induces corneal dysfunction, decreased vision, and ocular hypotonia. Effects of bufadienolides on the decrease of ocular pressure appear to be very strong, with quick action. This rarely described effect of the bufotoxin can be used as a basis for further research of toad venom and its pharmacological potential. *Purpose*. To present a case of a 67-year-old female patient who experienced a sudden decrease in vision after exposure to the poison from a common toad (*Bufo bufo*).

## 1. Introduction

The common toad, or European toad (*Bufo bufo*), is an amphibian found in almost all of Europe (with the exception of Iceland, Ireland, and some Mediterranean islands) and in parts of North Asia and North-west Africa. It can live up to 50 years in captivity, and its age is counted by growth rings on their phalanges [1]. It has green-gray-brown skin covered with lumps that produce bufadienolides. Bufadienolides are produced in parotid glands and the skin of a toad [2]. These toxic compounds are potent inhibitors of Na^+^/K^+^-ATPase activity, and toads use these as a natural repellent against predators and also as an immune defence against pathogens [3, 4]. Bufadienolides are divided into bufagenins, the smaller, hydrolysed molecules which have stronger cardiotoxic effects, and bufotoxins, the larger bufadienolide molecules with an amino-acid side chain [5]. Some research suggests that the ratio of bufadienolides depends of environmental factors [6]. Bufotoxin was first isolated by Hienrich Wieland in 1922, and its structure was described by the same team 20 years later (C_40_H_60_N_4_O_10_) [7]. The physiological effects of bufotoxin are similar to those of digitalis and causes increased heart rate and muscle contractions. Therefore, it is used worldwide in traditional medicine as an aphrodisiac [8] or as an anti-inflammatory agent [9]. Exposure to large amounts of toxin may cause cardiovascular and respiratory symptoms, such as paralysis and seizures, increased salivation, vomiting, hyperkalemia, cyanosis, and hallucinations [10]. Cases of poisoning of children were described after kissing a frog [11]. Ocular toxicity was also described [12, 13].

## 2. Case Report

A 67-year-old female patient was admitted to the emergency service because of sudden vision loss and a burning sensation in both eyes after she had been exposed to the poison of a common toad. The patient stated that she had been working in a garden when she noticed something moving under a cabbage leaf. She found a frog under the leaf, got scared, and instinctively stabbed the frog with a knife that she had in her hand. Right after she stabbed the frog, she got a spray of aerosol from the frog's skin on her face. She immediately felt a burning sensation in her eyes and eyelids, ran away to a washroom, and washed her face with a tap water. She noticed that her eyes were red and that her vision was very blurry.

One hour after, she had been examined by an ophthalmologist in a medical center. BCVA (*Best Corrected Visual Acuity*) was 0.3 in her right eye (RE) and 0.2 in the left eye (LE). Slit lamp examination showed conjunctival hyperaemia, slight oedema, and folds of the Descemet membrane of the cornea indicating ocular hypotonia. The anterior eye chamber was clear, and pupillary reactions were normal. Cataract was found on both eyes (grade CO2N1P0 in a LOCS III (*Lens Opacities Classification System*)). Hypotonia was confirmed by Goldmann aplanation tonometry with IOP (*intraocular pressure*) 9 mmHg in the right eye and 10 mmHg in the left eye. The fundus of both eyes was normal. After examination, eyes were washed with 0.9% saline solution and topical antibiotic ointment was administered (tobramicin 5 mg/4 times a day). After one hour, IOP increased to 10 mmHg in the right eye and 12 mmHg in the left eye. The patient was examined again after 6 hours, and regression of corneal oedema was found, as well as the improvement of vision; BCVA RE was 0.7 and BCVA LE was 0.5. IOP pressure also increased to 13 mmHg in both eyes. After 12 hours, BCVA improved to 0.8 in the right eye and 0.6 in the left eye. IOP pressure was 14 mmHg in the right eye and 15 mmHg in the left eye and Descemet membrane folds, as well as corneal oedema, were in remission.

## 3. Discussion

The toxic effect of bufadienolides on the cornea with consequential corneal oedema and ocular hypotension was probably caused by the inhibition of Na^+^/K^+^-ATPase, which is a well-known effect of the toad's venom [14]. Na^+^/K^+^-ATPase is a part of corneal endothelial cells and has an important role in maintaining corneal clarity through active pumping [15, 16]. It is also a part of the ciliary body epithelium and iris where it controls the rate of aqueous humor formation. It consists of *α*1 and *α*2 subunits which are present in the ciliary body epithelium. The *α*1 subunit is present on the basolateral surface of the pigmented epithelium, while the *α*2 subunit is located in the nonpigmented epithelium on the side facing the aqueous humor [17]. Some studies have suggested that the Na^+^/K^+^ ATPase *α*1 subunit might control overall sodium secretion to the aqueous humor, while the *α*2 may be responsible for the entry of sodium to the ciliary epithelium bilayer across the basolateral surface of the pigmented epithelium [18–20]. Inhibition of Na^+^/K^+^-ATPase in these tissues caused by exposure to bufadienolides was the most obvious reason for corneal dysfunction that caused a transient decrease of vision, and also for a hypotonia as a result of ciliary body dysfunction and disturbed control of aqueous humor production. The improvement in IOP after a few hours was probably not caused by the administered topical antibiotic treatment but rather by time passed from the exposure, where the irrigation of the ocular surface helped to remove the remaining toxin and inhibited continuous exposure, which resulted in ciliary function recovery and IOP improvement.

In conclusion, the poison of a common toad can cause ocular hypotonia and corneal oedema. Effects of bufadienolides on a decrease of ocular pressure appear to be very strong, with quick action and caused by Na^+^/K^+^ ATPase inhibition in corneal endothelium cells, ciliary body, and iris. This rarely described effect of bufadienolides could be used as a basis for further research of a toad's venom and its effects on ocular surface and pharmacological potential.
